# Dissecting cellulitis of the scalp in a paediatric male

**DOI:** 10.1111/ajd.14342

**Published:** 2024-06-24

**Authors:** Meryl Thomas, Valerie Yii, Rodney Sinclair

**Affiliations:** ^1^ Sinclair DIRECT, Dermatology Investigational Research, Education and Clinical Trials Centre Melbourne Victoria Australia; ^2^ Monash School of Medicine, Monash University Melbourne Victoria Australia; ^3^ Epworth Healthcare Melbourne Victoria Australia

## PATIENT CONSENT

Written consent provided by next of kin.

**FIGURE 1:**
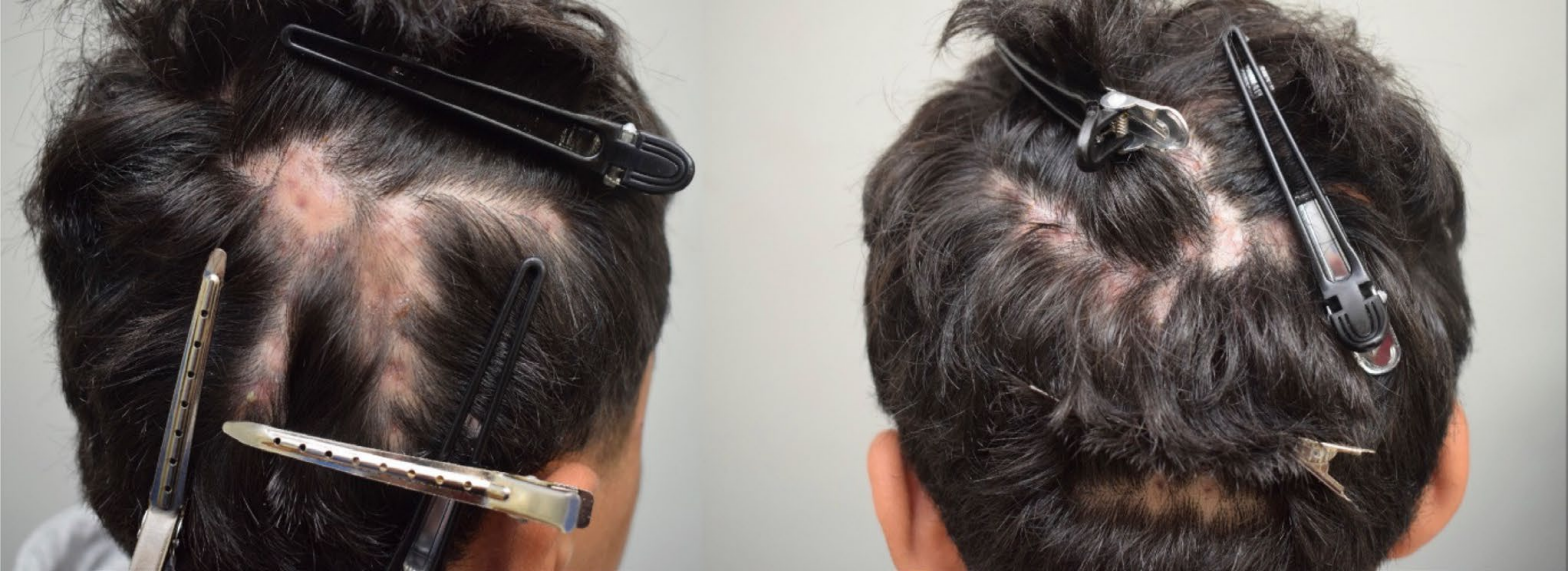
Vertex, occipital and parietal scalp findings when the patient first presented to the hair clinic. Patches of hair loss, nodules and pustules characteristic of dissecting cellulitis of the scalp are seen.

Dear Editor,

A 13‐year‐old boy of Iraqi heritage presented with alopecia and scalp nodules, refractory to doxycycline, clindamycin and topical steroids. There was no significant past medical history. He has a maternal uncle with alopecia areata. Examination revealed multiple tender, erythematous nodules overlying the areas of alopecia. In addition, there were a few scattered pustules (Figure 1). While he had mild acne vulgaris on his forehead, there was no evidence of acne conglobata, hidradenitis suppurativa or pilonidal sinus. Swab from a scalp pustule did not show any bacterial or fungal growth.

**FIGURE 2:**
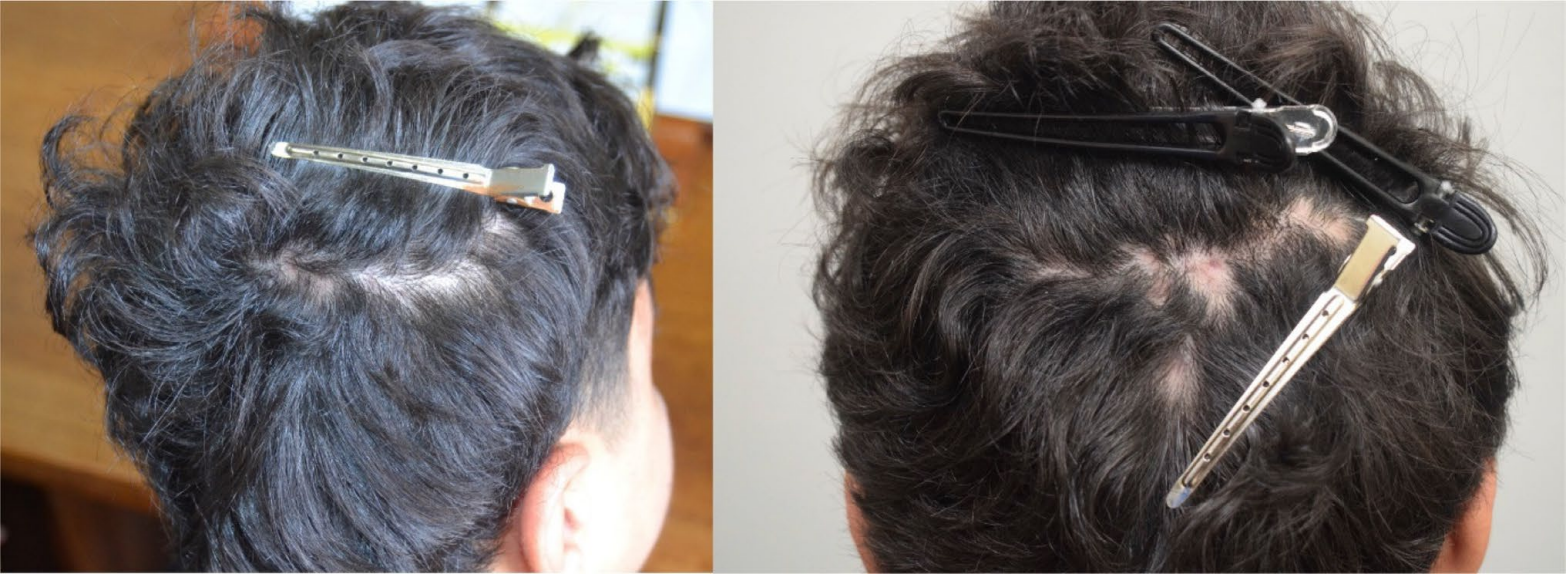
Ten weeks after commencing treatment, there are no active inflammatory lesions and there is some regrowth in the areas of patchy hair loss.

The clinical diagnosis was dissecting cellulitis of the scalp. The patient was commenced on isotretinoin 10 mg daily (0.20 mg/kg), sublingual minoxidil 0.45 mg twice daily, oral prednisolone 25 mg daily for 2 weeks, then 12.5 mg daily for 4 weeks and antiseptic shampoo. On review 4 weeks later, the inflammatory scalp lesions had flattened; no new nodules or pustules had appeared and there was significant hair regrowth within some but not all the scalp patches. Further improvement was noted 10 weeks after initiation of treatment, and prednisolone was reduced to 10 mg daily (Figure 2). Apart from increased hair growth on the upper lip, there were no other side effects from the treatment regime.

Dissecting cellulitis of the scalp (DCS) is an inflammatory dermatosis of the scalp,^1^ characterised by suppurative and often interconnected nodules that can cause cicatricial alopecia.^2^ The condition may be associated with acne conglobata, hidradenitis suppurativa and pilonidal sinus.^1^ Lesions are most commonly seen on the vertex of the scalp.^3^ DCS is most common in African–American men between the ages of 20 and 40 years,^1^ though it has also been reported in women, adolescents^1^ and in multiple ethnic groups.^3^ Smoking is thought to increase risk of DCS.^4^ The condition has been reported in a 10‐year‐old girl.^5^ It has also been documented in a 15‐year‐old boy.^6^ To the best of our knowledge, this is the youngest reported case of DCS in a male.

Treatment of DCS is largely empirical as there have been no randomised controlled trials for this rare scalp condition. A systematic review of available treatments recommended isotretinoin first line. Dosages ranging from 0.25 mg/kg/day to 1 mg/kg/day have been documented,^7^ though as displayed in this case, even lower doses may be sufficient. Skeletal abnormalities have been reported in children receiving prolonged treatment over several years with oral retinoids; however, short‐term use does not appear to have any substantial effects on bone mineralisation.^8^ For DCS resistant to oral retinoids, biologic agents can be considered.^9^ Periodic nodule aspiration may be beneficial^9^ and severe cases may require wide local excision of the affected area, resulting in permanent alopecia.^1^

A short course of oral steroids was used in this case as an adjunct to isotretinoin to control the active inflammation, halt nodule formation and reduce the risk of scarring. Intralesional corticosteroid injection may also be used.^1^ Minoxidil was commenced to accelerate hair regrowth. A systematic review of minoxidil in scarring alopecias found benefit in terms of disease stabilisation and hair regrowth.^10^ The sublingual formulation theoretically provides greater bioavailability than oral.^11^

Though uncommon in adolescents, DCS should be considered in paediatric patients with scalp nodules and patchy hair loss. The mainstay of treatment in the paediatric and adult population is isotretinoin. Minoxidil is a useful adjunct to augment regrowth.

## FUNDING INFORMATION

None.

## CONFLICT OF INTEREST STATEMENT

Professor Rodney Sinclair is an Editorial Board member of Australasian Journal of Dermatology and a co‐author of this article. To minimise bias, they were excluded from all editorial decision‐making related to the acceptance of this article for publication.

## Data Availability

Data sharing not applicable to this article as no datasets were generated or analysed during the current study.

## References

[1] Nussbaum D , Desai S , Nelson K , Saardi K , Friedman A . An up‐to‐date approach to the management of dissecting cellulitis. J Drugs Dermatol. 2022;21(7):800–802. 10.36849/JDD.0421 35816064

[2] Guo W , Zhu C , Stevens G , Silverstein D . Analyzing the efficacy of Isotretinoin in treating dissecting cellulitis: a literature review and meta‐analysis. Drugs R&D. 2021;21(1):29–37. 10.1007/s40268-020-00335-y PMC7937584 33387328

[3] Lee C‐N , Chen W , Hsu C‐K , Weng T‐T , Lee JY‐Y , Yang C‐C . Dissecting folliculitis (dissecting cellulitis) of the scalp: a 66‐patient case series and proposal of classification. J Dtsch Dermatol Ges. 2018;16(10):1219–1226. 10.1111/ddg.13649 30168900

[4] Tran AX , Lefante JJ , Murina A . Risk factors for dissecting cellulitis of the scalp: a case‐control study. J Am Acad Dermatol. 2022;86(4):941–943. 10.1016/j.jaad.2021.03.076 33785386

[5] Ramesh V . Dissecting cellulitis of the scalp in 2 girls. Dermatologica. 1990;180(1):48–50. 10.1159/000247985 2106459

[6] Arneja JS , Vashi CN , Gursel E , Lelli JL . Management of fulminant dissecting cellulitis of the scalp in the pediatric population: case report and literature review. Can J Plast Surg. 2007;15(4):211–214. 10.1177/229255030701500406 19554179 PMC2696005

[7] Melo DF , Trüeb RM , Dutra H , Lima M , Machado CJ , Dias M . Low‐dose isotretinoin as a therapeutic option for dissecting cellulitis. Dermatol Ther. 2020;33(6):e14273. 10.1111/dth.14273 32890448

[8] Margolis DJ , Attie M , Leyden JJ . Effects of isotretinoin on bone mineralization during routine therapy with isotretinoin for acne vulgaris. Arch Dermatol. 1996;132(7):769–774. 10.1001/archderm.1996.03890310053007 8678568

[9] Masson R , Jeong CY , Ma E , Crew AB , Fragoso NM , Shi VY , et al. Treatments for dissecting cellulitis of the scalp: a systematic review and treatment algorithm. Dermatol Ther. 2023;13(11):2487–2526. 10.1007/s13555-023-01018-7 PMC10613185 37740150

[10] Ly N , McClure EM , Hordinsky MK , Farah RS , Park SY . Safety and efficacy of Minoxidil treatment in scarring alopecia: a scoping review. J Drugs Dermatol. 2024;23(3):146–151. 10.36849/jdd.7743 38443124

[11] John JM , Sinclair RD . Systemic minoxidil for hair disorders in pediatric patients: a safety and tolerability review. Int J Dermatol. 2023;62(2):257–259. 10.1111/ijd.16373 35965281

